# Candida Endocarditis in Patients with Candidemia: A Single-Center Experience of 14 Cases

**DOI:** 10.1007/s11046-020-00492-3

**Published:** 2020-10-09

**Authors:** Florian Hitzenbichler, Tobias Joha, Michaela Simon, Jirka Grosse, Karin Menhart, Dirk Hellwig, Daniele Camboni, Sabine Sag, Can Martin Sag, Frank Hanses, Bernd Salzberger, Arno Mohr

**Affiliations:** 1 Department of Infection Prevention and Infectious Diseases, University Hospital Regensburg, Franz-Josef-Strauß-Allee 11, 93053 Regensburg, Germany; 2 Institute of Clinical Microbiology and Hygiene, University Hospital Regensburg, Franz-Josef-Strauß-Allee 11, 93053 Regensburg, Germany; 3 Department of Nuclear Medicine, University Hospital Regensburg, Franz-Josef-Strauß-Allee 11, 93053 Regensburg, Germany; 4 Department of Cardiothoracic Surgery, University Hospital Regensburg, Franz-Josef-Strauß-Allee 11, 93053 Regensburg, Germany; 5 Department of Internal Medicine II, University Hospital Regensburg, Franz-Josef-Strauß-Allee 11, 93053 Regensburg, Germany

**Keywords:** *Candida*, Candidemia, Endocarditis, ^18^F-FDG PET/CT, Echinocandins, Outcome

## Abstract

A retrospective, single-center analysis of 14 cases of *Candida* endocarditis (from 355 candidemia cases during the years 2012–2019) revealed a high in-hospital mortality (57.1%), a high proportion of healthcare-associated infections (13/14) and a high treatment preference for echinocandins. Transthoracic echocardiography and ^18^F-FDG PET/CT had a sensitivity of 54.5% and 57.1%, respectively. Patients were older than previously described and most patients with Candida endocarditis had persistent candidemia for ≥ 3 days despite antifungal therapy.

**Electronic supplementary material:**

The online version of this article (10.1007/s11046-020-00492-3) contains supplementary material, which is available to authorized users.

## Introduction

An increase in candidemia and in *Candida* endocarditis is expected due to rising numbers of patients with immunosuppression and intravascular or intracardiac devices [1, 2].

Only 2% of endocarditis cases are of fungal origin, but mortality in these patients is high and diagnosis is complicated due to a low sensitivity of blood cultures (BC) for *Candida* spp. [2].

In this case series, we describe our experience with *Candida* endocarditis (CE) in patients who were treated at our hospital in a 14-year period.

## Patients and Methods

Regensburg University Hospital is an 839-bed tertiary care academic teaching hospital in Germany.

All reports on BCs positive for *Candida* spp. between January 1, 2006 and December 31, 2019 were retrieved from the microbiology laboratory database and patients’ charts were reviewed retrospectively by three of the authors (TJ, AM, FHi).

Patients were classified as having CE, if *one* of the following two conditions applied:

1. Positive *Candida* culture *or* histopathological findings consistent with fungal endocarditis from a surgically resected heart valve.
2. Echocardiographic evidence of endocardial involvement (in patients with candidemia).

All identified cases were reviewed independently by two of the authors (AM, FHi) for plausibility (where all available data including discharge letters and discharge diagnosis were taken into account).

Patients were considered to have healthcare-acquired candidemia if at least one of the following conditions applied [3]:

1. First positive *Candida* BC ≥ 48 h after admission to an acute care hospital.
2. Admission from a long-term care facility or rehabilitation hospital with candidemia.
3. Attendance of a dialysis clinic in the last 30 days before admission with candidemia.

Persistent candidemia was defined as a BC positive for the same *Candida* species on ≥ 3 days (72 h) despite initiation of antifungal therapy, which was similar to a definition previously published [4].

Species differentiation and antifungal susceptibility testing of *Candida* isolates were performed according to local laboratory standards (detailed information in the Supplement.)

In some patients, ^18^F-FDG PET/CT (PET/CT) imaging was performed. (Detailed information on the PET/CT protocol performed at our hospital in the Supplement.) All available PET/CT images were reviewed retrospectively by one of the authors (JG), who was aware that CE was diagnosed in these patients.

PET/CT was not available in all patients of our study since it is not routinely performed in patients with endocarditis at our hospital.

The analysis was approved by the local ethics committee (No. 18-1240-104, waiver due to the retrospective nature of the study).

## Results

Between January 1, 2012 and December 31, 2019 355 cases of candidemia were recorded at our hospital. 15 patients were judged to have CE according to our definition (4.2%). Before 2012 no cases of CE could be documented at our hospital (years 2006–2011; *n* = 165 cases of candidemia), so these years were excluded from our analysis.

**Fig. 1:**
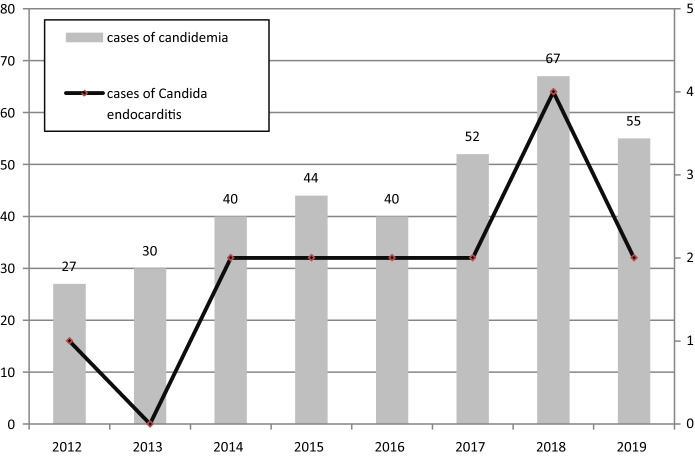
Cases of candidemia and Candida endocarditis over the time period 2012–2019. Before 2012 (2006–2011) no cases of Candida endocarditis could be identified retrospectively

The distribution of cases with CE and candidemia over time is shown in Fig. 1. Detailed information on the 14 included patients is listed in Table 1.

**Table 1:** Detailed characteristics of all 14 patients with Candida endocarditis during the study period (2006–2019)

Patient number	Gender/age	Case synopsis	Infection site	*Candida* spp. MIC [mg/L]	Time between first cardiac surgery and candidemia in months	Vegetation size (TEE)/visible on TTE also	PET/CT result (time between begin therapy and PET in days)	Other Foci in PET/CT	Therapy surgical therapy/antifungals	Time between first positive and first negative BC in days	Outcome
1	F/86	Admitted with *E. faecalis* endocarditis, candidemia during hospital stay (CVC), Candida and Enterococcus both cultured from operated valve	Native mitral valve	*C. albicans* Fluconzole **S** [0.25] Anidulafungin **NA** AmpB **NA**	–	17 mm/yes Severe MR	NA	–	Valve replacement/Fluconazole 400 mg first (11 days) then switch to Caspofungin 70 mg	1 (only one positive BC for Candida)	Discharged no information available thereafter
2	M/46	Admitted with Hodgkin´s lymphoma Two episodes of candidemia during hospital stay (CVC) diagnosis of endocarditis during second episode Candida cultured from explanted valve	Bicuspid aortic valve	*C. albicans* Fluconazole **R** [12] Anidulafungin **S** [0.004] AmpB **S** [0.38]	–	17 mm/no	No increased FDG uptake in area of aortic valve (11)	None	Valve replacement/Anidulanfungin 100 mg first then switch to AmpB 5 mg/kg	No negative BC (no clearance)	Deceased during hospital stay
3	M/63	Admitted with endocarditis after cardiac surgery Most likely hospital-acquired (CVC)	Prosthetic aortic valve (biological) *and* mitral valve reconstruction	*C. albicans* Fluconazole **S** [0.38] Anidulafungin **S** [0.006] AmpB **NA**	4	13 mm/yes Severe MR &AR/	NA	–	None/Combination of Fluconazole 200 mg and Caspofungin 50 mg	no negative BC (no clearance)	Deceased during hospital stay
4	F/74	Admitted to local hospital with cerebral hemorrhage, after diagnosis of candidemia referral to our hospital; candidemia most likely hospital-acquired (CVC) Candida cultured from explanted valve	Mitral valve reconstruction	*C. albicans* Fluconazole **S** [0.38] Anidulafungin **S** [0.002] AmpB **NA**	3	12 mm/no	Increased FDG uptake in area of mitral valve (6)	None	Valve replacement/Combination of Caspofungin 70 mg and Fluconazole 400 mg, recommendation to indefinite oral therapy with Fluconazole 400 mg	7	Discharged no information available thereafter
5	M/67	Admitted with *Granulicatella* endocarditis, after valve replacement discharge, 2 months later re-admission with candidemia, most likely hospital-acquired (CVC) Candida cultured from explanted valve	Mitral valve reconstruction	*C. albicans* Fluconazole **S** [0.38] Anidulafungin **S** [0.002] AmpB **NA**	2	20 mm/yes Severe MR	Increased FDG uptake in area of mitral valve *in retrospective analysis only* (8)	Brain, Spleen	Valve replacement/Combination of AmpB 5 mg/kg and Caspofungin 70 mg	14	Deceased during hospital stay
6	M/63	Referral with candidemia from external hospital, renal insufficiency and Myasthenia gravis Candidemia most likely hospital-acquired (dialysis) PET/CT confirmed diagnosis of endocarditis before TEE (later positive for vegetations as well)	Prosthetic aortic valve (biological)	*C. albicans* Fluconazole **S** [0.25] Anidulafungin **S** [0.004] AmpB **NA**	2	2 mm/no	Increased FDG uptake in area of aortic valve (3)	None	None/Combination of Caspofungin 70 mg and Fluconazole 400 mg and after 30 dys AmpB 5 mg/kg	30	Deceased during hospital stay
7	F/68	Admitted with abnormal TEE Candidemia during hospital stay most likely hospital-acquired (CVC)	Prosthetic aortic valve (biological) + aortic prosthesis	*C. albicans* Fluconazole **S** [0.25] Anidulafungin **S** [0.003] AmpB **NA**	11	7 mm/yes	Increased FDG uptake in area of aortic prosthesis and aortic valve (1)	None	None/Caspofungin 150 mg	3	Discharged, no information available thereafter
8	F/79	Admitted with NSTEMI, after Coronary artery bypass surgery candidemia (hospital-acquired, CVC)	Native aortic *and* tricuspid valve	*C. albicans* Fluconazole **S** [1] Anidulafungin **S** [0.003] AmpB **S** [0.25]	–	11 mm/NA	NA	–	None/Combination of Caspofungin 50 mg and Fluconazole 400 mg and after 14 dys AmpB 5 mg/kg	21	Discharged to other hospital, deceased 8 days after discharge
9	M/82	Admitted with *S. haemolyticus* prosthetic valve endocarditis, after valve replacement candidemia (hospital-acquired, CVC)	Prosthetic aortic valve (biological)	*C. albicans* Fluconazole **S** [0.2] Anidulafungin **S** [0.002] AmpB **NA**	< 1 (13 days)	3 mm/yes	No increased FDG uptake in area of aortic valve (7)	LWK 2 Lungs	None/Caspofungin 50 mg first, then step down to Fluconazole 400 mg	No negative BC (no clearance)	Discharged, last date known to be alive 8 months after discharge
10	F/83	Admitted with cardiac decompensation Respiratory failure and NIV therapy candidemia hospital-acquired (CVC)	LAA Occluder	*C. tropicalis* Fluconazole **S** [0.012] Anidulafungin **S** [0.002] AmpB **NA**	NA	13 mm/NA Severe MR	NA	–	None/Caspofungin 50 mg	3	Deceased during hospital stay
11	F/65	Admitted with port candidemia, Hodgkin´s lymphoma III Healthcare-associated candidemia (port)	Native tricuspid valve	*C. albicans* Fluconazole S [0.25] Anidulafungin S [0.002] AmpB NA	–	5 mm/no	NA	–	None/Caspofungin 35 mg first, then step down to Fluconazole 400 mg after 25 dys	10	Discharged, no information available thereafter
12	F/65	Admitted with decompensated liver cirrhosis, candidemia hospital-acquired (CVC)	Native aortic valve	*C. albicans* Fluconazole **S** [0.5] Anidulafungin **S** [0.002] AmpB **NA**	–	6 mm/NA Severe TR	NA	–	None/Caspofungin 35 mg first, then step down to Fluconazole 200 mg after 50 dys, Indefinite treatment recommended	2	Discharged, no information available thereafter
13	F/83	Admitted with clinical deterioration During hospital stay candidemia (hospital-acquired), valve replacement and AICD removal, hardware not sent for culture	Prosthetic aortic valve (biological)/AICD	*C. albicans* Fluconazole **S** [0.38] Anidulafungin **S** [0.003] AmpB **NA**	3	15 mm/yes Severe MR perivalvular abscess	NA	–	AICD removal/Combination of Caspofungin 50 mg and Fluconazole 400 mg	7	Deceased during hospital stay
14	M/74	Admitted with catheter-associated infection (port) Patient with liver cirrhosis and hepatocellular carcinoma Hospital-associated infection (chemotherapy)	Native tricuspid valve	*C. glabrata* Fluconazole R [> 256] Anidulafungin S [0.016] AmpB S **NA**	–	Very small/no	No increased FDG uptake in area of tricuspid valve	None	Caspofungin 100 mg	1 (only one positive BC for *Candida* sp.)	Deceased during hospital stay

*F* female, *M* male, *CVC* Central venous catheter, *CE* Candida endocarditis, *AICD* automatic implantable cardioverter-defibrillator, *AmpB* Amphotericin B, *S* susceptible, *R* resistant, *MIC* Minimum inhibitory concentration, *TEE* transesophageal echocardiography, *TTE* transthoracic echocardiography, *NA* not available, *PET/CT* ^18^F-FDG PET/CT, *BC* blood culture, *MR* mitral valve regurgitation, *AR* aortic valve regurgitation, *TR* tricuspid valve regurgitation

One (female) patient had to be excluded from our analysis due to missing data. The patient was only seen in our emergency department, where in transesophageal echocardiogram (TEE) the diagnosis of endocarditis was made (previous valve surgery) and *Candida tropicalis* was isolated in three different BC sets. However, the patient was transferred to another university hospital after TEE (and before BCs were available) and is therefore lost for follow-up.

### Clinical Characteristics

Eight of 14 patients were female (57.1%), the median age was 71 years (range 46–86 years). Median Charlson score was 4 (range 2–8).

A total of 13 episodes of candidemia were healthcare-acquired with a central venous catheter (CVC) being the most likely source of fungemia in ten patients and a port catheter in two other patients (No. 11 & 14). Twelve patients had previous intravenous antibiotic therapy.

Median hospital stay was 39 days (range 33–130 days).

### Microbiology

In twelve patients *C. albicans* was isolated. Other species (*C. parapsilosis, C. glabrata)* were found in only two patients (No. 10 & 14). Only two isolates were fluconazole resistant (*C. albicans*, patient No. 2, with previous fluconazole therapy; *C. glabrata* in patient No. 14 with intrinsic fluconazole resistance). No isolate was resistant to anidulafungin (one not tested, MIC range 0.02–0.06 mg/L).

Eleven patients (78.6%) had persistent candidemia according to our definition.

### Diagnostics, Treatment and Outcome

In 12/14 patients an ophthalmological evaluation was available, involvement of the eye could be documented in only three patients (No. 2, 9, 14). Six patients had septic brain emboli (diagnosed in MRI); three patients had septic peripheral embolization (as diagnosed in CT scan and PET/CT). In 4/7 (57.1%) patients, PET/CT confirmed endocarditis (PET/CT was available only in seven patients of our study).Vegetation size in TEE was > 10 mm in eight patients (*median* 12 mm).

Infectious diseases consultation was requested in all patients at some point.

Echinocandins were used in all except one patient (No. 1) primarily. In six cases antifungal combination therapy was used, combining echinocandins with either liposomal amphotericin B or fluconazole. Three patients were switched from an echinocandin to liposomal amphotericin B due to various reasons (persistent candidemia, cerebral manifestation of CE).

Five patients received surgical treatment (valve replacement, removal of AICD), in all other cases operation was not judged feasible by the treating surgeon. Chronic suppressive therapy was discussed on a case-by-case basis and recommended in two cases. Median time interval between first candidemia episode and surgery was 9 days (range 4–20 days).

Eight patients died during their hospital stay (mortality rate: 57.1%).

## Discussion

4.2% of patients with candidemia had CE—similar to a recent study where CE was diagnosed in 4.2% of patients with candidemia also [5]. Persistent candidemia was frequent (84.6%) as was healthcare-acquisition (92.3%). Previous studies found a rate of healthcare-associated CE between 65 and 90% [3, 4, 6, 7].

The increase in CE rates over the years is surprising. However, it must be taken into account that in previous years guidelines on management of candidemia were different and echocardiography was not routinely recommended. In a recent study, we could demonstrate an increase of candidemia cases in general over the last years (which might obviously lead to more cases of CE). This might be due to an increase in sicker and older patients during the recent years at our hospital. Furthermore, TEE is performed more often in patients with candidemia at our hospital (in 11.6% of patients with candidemia in 2006–2008 vs. 40.3% in 2016–2018), which might also lead to a higher rate of diagnosed CE [8].

Only one of the patients described (No. 2) was younger than 60 years, all other patients were between 63 and 86 years old, which is different to other studies where the study population is slightly younger (median age: 55 years [7], 66 years [3], 59 years [2], mean age: 54 years [4]) In the pathological study of Vaideeswar et al. [6], patients were considerably younger (mean age: 23 years).

In 8/14 patients, a history of cardiac surgery was present. Nearly all patients after prosthetic valve insertion or valve reconstruction developed CE within the first 6 months after surgery. In the study of Rivoisy and colleagues, the median time between surgery and CE was 8 months [3].

All prosthetic valves in our analysis were of biological origin. Similarly, Antinori et al. [2] found biological valves in all four patients with prosthetic valve CE. A recent meta-analysis observed CE in both patients with biological (*n* = 31) and mechanical valves (*n* = 25) [1]. Patients with biological heart valves developed CE significantly earlier after surgery (< 1a) than patients with mechanical valves. The authors speculated that formation of fungal biofilm requires some sort of biological component (either biological grafts or neoendocardium in mechanical valves) which might contribute to the time difference in development of CE after surgery (since endothelialisation of mechanical valves may take up to 24 months). Other studies did not differentiate between mechanical and biological prosthetic valves [4, 6, 7].

Large heart valve vegetations were found on TEE in most patients (*median* size in this study: 12 mm) and transthoracic echocardiogram (TTE) was not reliable in detecting vegetations (sensitivity 54.5%). Large vegetations seem to be frequent in CE (in other studies: *median* size: 17 mm [3], 15 mm [7]).

PET/CT was not able to confirm CE in three patients despite of a large vegetation (17 mm) in one of them (No. 2). The same patient (No. 2), however, had leucopenia during the examination (leucocyte count 1.4/nl, 70% neutrophils). In recent studies, sensitivity of PET/CT was not compromised in patients with febrile neutropenia, but data on PET/CT in patients with invasive fungal disease *and* neutropenia are scarce [9]. A French study reported 83% sensitivity of PET/CT in invasive candidiasis (liver, spleen, bone infections), but patients with CE were not included [10]. The low sensitivity compared to other studies that specifically evaluated the sensitivity of FDG PET/CT in endocarditis may also be due to the fact that patients in our study did not receive a PET/CT scan with the intention to diagnose endocarditis, but for the detection of an infectious focus in general. Thus, suppression of myocardial nuclide uptake, e.g., by a low-carbohydrate diet or intravenous preadministration of heparin, was not routinely performed.

To the best of our knowledge, this is the first series where sensitivity of PET/CT in CE was analyzed, but due to the small sample size, our results are still preliminary. Sensitivity is low in our cohort and comparable to TTE, however, the strength of PET/CT scans in patients with endocarditis might be in diagnosis of other foci or septic emboli or to confirm endocarditis in uncertain cases.

Echinocandins were used as first line therapy in 13/14 patients in this analysis. Therapy was maintained if clearance of candidemia could be achieved; in all other cases combination therapy was used. Recent guidelines recommend either liposomal amphotericin B or higher-dosed echinocandins for CE [11].

In a retrospective analysis of 46 patients with prosthetic valve, CE patients receiving liposomal amphotericin B had a better outcome than those receiving monotherapy with an echinocandin [3]. In another recent study, patients with (both native and prosthetic valve) CE treated with an echinocandin had a similar outcome to those receiving liposomal amphotericin B despite a higher percentage of older patients and nosocomial infections in the echinocandin group [4]. In both studies, dosing regimens for echinocandins were not reported. The recent ESC guidelines for the management of infective endocarditis recommend to use echinocandins at higher doses also [12]. In only two patients of our study, higher doses of caspofungin were used, where no relevant side effects were noted. In some patients, higher doses of echinocandins were avoided due to comorbidities (like liver disease) or treatment was changed to combination therapy after infectious diseases consultation.

However, the role of combination therapy still remains unclear. Despite early use of echinocandins and a high rate of combination therapy, mortality in our series was 57%. In two retrospective studies, a similar high mortality was seen [3, 7], other studies report a lower in-hospital mortality of below 40% [1, 4].

This study has the following limitations:

1. Since strict criteria for diagnosis of CE were applied, this might underestimate the true incidence of CE in this cohort, which might even be higher, since TEE is not always reliable for diagnosis of endocarditis. However, we tried to accommodate this problem by doublechecking for the diagnosis of endocarditis in the discharge letters. We were, however, not able to correlate our cohort with all patients with culture-negative endocarditis in the same period, since we were not able to retrieve all these patients from our data base. The same applies for patients with persistent candidemia (and without diagnosis of CE in discharge letters).
2. Standard incubation time of BCs was 5 days (*supplement*). This might underestimate incidence of CE, since Candida diagnostic in BC is known to be limited with decreases sensitivity [11].
3. We were not able to provide drug levels of antifungals in our manuscript, since therapeutic drug monitoring (TDM) for fluconazole, echinocandins or liposomal amphotericin B is not available at our hospital.

In summary, Candida endocarditis is a rare condition (occurring in about 4% of patients with candidemia in our recent series) and cases are frequently healthcare-acquired. Risk factors include older age (> 60 years) and immunosuppression. Nearly all patients with CE had persistent candidemia. CE affected both native and prosthetic valves and should be considered an early event (mostly during the first 6 months) after biological heart valve surgery or valve reconstruction. Both PET/CT and TTE had a low sensitivity in diagnosis of CE (< 60%). The optimal treatment is still a matter of debate, but echinocandins are being more frequently used as first line therapy (maybe due to better tolerability). Mortality is still alarmingly high (clearly above 50%).

## Electronic supplementary material

Below is the link to the electronic supplementary material.

Supplementary material 1 (DOCX 15 kb)

## Data Availability

Data not publically available.
