# Supplementary material for: Candida Endocarditis in Patients with Candidemia: A Single-Center Experience of 14 Cases
Source: Mycopathologia. 2020 Oct 9;185(6):1057–67. doi: 10.1007/s11046-020-00492-3 (PMC7779412; doi:10.1007/s11046-020-00492-3)
Supplement: Supplementary file 1 — Supplementary material 1 (DOCX 15 kb) [file 11046_2020_492_MOESM1_ESM.docx]

**Supplemental Material**

for **Candida endocarditis in patients with candidemia - a single center experience of 14 cases**

*Methods - microbiology*

Growth of *Candida* spp. was detected with the BACTEC automated blood culture system (Becton Dickinson, Heidelberg, Germany) using the BACTEC 9240 device. In 2011, it was replaced with the BACTEC FX device. Blood cultures were performed with the BACTEC Plus Aerobic medium and the Standard Anaerobic medium, which was replaced with the Lytic Anaerobic medium in 2017. Standard incubation time was five days. Identification of the cultured isolates was accomplished using the API *Candida* biochemical test strips (Biomérieux, Marcy-l'Étoile, France France). From 2011 onwards, isolates were identified with matrix-assisted laser desorption ionization-time of flight mass spectrometry (MALDI TOF MS) using the Microflex mass spectrometer with the BioTyper software (Bruker Daltonics, Bremen, Germany). Minimal inhibitory concentrations (MIC) were determined with Etest gradient strip tests on RPMI MOPS Glucose agar plates (Biomérieux, Marcy-l'Étoile, France). The MICs were interpreted according to clinical breakpoints provided by CLSI, from 2014 onwards EUCAST breakpoints were applied.

*Methods - echocardiography*

Transthoracic or transesophageal echocardiography was performed using a standard ultrasound system by Philips (Philips iE33 or Philips sparq).

*Methods – PET/CT protocol*

In three patients a Biograph 16 PET/CT scanner (CTI-Siemens, Erlangen, Germany) consisting of a 16-slice multidetector CT (0.5 s per revolution) was used; in three patients a Biograph mCT 40 FLOW PET/CT scanner (CTI-Siemens, Erlangen, Germany), consisting of a 40-slice multidetector CT (0.5 s per revolution) was used.

After a fasting period of at least 6 h, 3 MBq ^18^F-FDG per kilogram body weight were injected intravenously (254±43 MBq). The patients’ blood glucose level was strictly controlled to be below 150 mg/dL (8.32 mmol/L). To increase renal tracer elimination, patients received an injection of 20 mg furosemide as well as intravenous hydration shortly after ^18^F-FDG injection.

In order to minimize muscular ^18^F-FDG uptake, patients were advised to stay in a quiet lying position. Warming blankets were used to avoid freezing of the patients and to keep potential tracer accumulation in brown fat tissue to a minimum. Patients were instructed to void the bladder prior to scanning and to remove all metal parts.

After a waiting period of about 60 min post-injection (patient No. 6 only 45 min), the PET/CT acquisition was performed. Using the Biograph 16 PET/CT scanner, images of the trunk were acquired with elevated arms (pelvis to skull or skull base). Depending on the patient size and clinical indication, six to eight overlapping bed positions with 3 min of PET acquisition time each were used. Using the Biograph mCT 40 FLOW PET/CT scanner, images of the whole body (skull to feet) were acquired using the continuous bed move (torso: 0,8 cm/min, legs: 1,1 cm/min). The same area was covered by a low-dose CT scan (tube current 50 mAs, tube voltage 120 keV). No contrast agents were given.

PET images (slice thickness 5 mm) were corrected for random coincidences, decay, scatter, and attenuation and reconstructed iteratively using the ordered subsets expectation maximization algorithm (OSEM) with four iterations and eight subsets. PET images were scaled to allow SUV measurements. PET and CT images were checked for breathing artifacts. Attenuation-corrected and uncorrected images were evaluated. Both data sets were routinely analyzed and compared. PET positivity was evaluated by visual and quantitative determination (measurement of the SUV) of the ^18^F-FDG uptake, pattern and localization. A suppression of the myocardial nuclide uptake was not routinely performed, since the patients were referred to the Department of Nuclear Medicine for the detection of an infectious focus in general.

*Methods – other diagnostics*

Routinely in all patients with endocarditis MR scan of the brain is performed. Diagnosis of other septic emboli is guided by symptoms, but usually a CT scan (or at least abdominal ultrasound) is performed routinely in patients with endocarditis.
